# Reversing Melanoma Cross-Resistance to BRAF and MEK Inhibitors by Co-Targeting the AKT/mTOR Pathway

**DOI:** 10.1371/journal.pone.0028973

**Published:** 2011-12-14

**Authors:** Mohammad Atefi, Erika von Euw, Narsis Attar, Charles Ng, Connie Chu, Deliang Guo, Ramin Nazarian, Bartosz Chmielowski, John A. Glaspy, Begonya Comin-Anduix, Paul S. Mischel, Roger S. Lo, Antoni Ribas

**Affiliations:** 1 Department of Medicine, Division of Hematology/Oncology, University of California Los Angeles, Los Angeles, California, United States of America; 2 Department of Pathology and Laboratory Medicine, University of California Los Angeles, Los Angeles, California, United States of America; 3 Department of Medicine, Division of Dermatology, University of California Los Angeles, Los Angeles, California, United States of America; 4 Department of Surgery, Division of Surgical Oncology, University of California Los Angeles, Los Angeles, California, United States of America; 5 Jonsson Comprehensive Cancer Center at University of California Los Angeles, Los Angeles, California, United States of America; University Hospital Hamburg-Eppendorf, Germany

## Abstract

**Background:**

The sustained clinical activity of the BRAF inhibitor vemurafenib (PLX4032/RG7204) in patients with BRAF^V600^ mutant melanoma is limited primarily by the development of acquired resistance leading to tumor progression. Clinical trials are in progress using MEK inhibitors following disease progression in patients receiving BRAF inhibitors. However, the PI3K/AKT pathway can also induce resistance to the inhibitors of MAPK pathway.

**Methodology/Principal Findings:**

The sensitivity to vemurafenib or the MEK inhibitor AZD6244 was tested in sensitive and resistant human melanoma cell lines exploring differences in activation-associated phosphorylation levels of major signaling molecules, leading to the testing of co-inhibition of the AKT/mTOR pathway genetically and pharmacologically. There was a high degree of cross-resistance to vemurafenib and AZD6244, except in two vemurafenib-resistant cell lines that acquired a secondary mutation in NRAS. In other cell lines, acquired resistance to both drugs was associated with persistence or increase in activity of AKT pathway. siRNA-mediated gene silencing and combination therapy with an AKT inhibitor or rapamycin partially or completely reversed the resistance.

**Conclusions/Significance:**

Primary and acquired resistance to vemurafenib in these *in vitro* models results in frequent cross resistance to MEK inhibitors, except when the resistance is the result of a secondary NRAS mutation. Resistance to BRAF or MEK inhibitors is associated with the induction or persistence of activity within the AKT pathway in the presence of these drugs. This resistance can be potentially reversed by the combination of a RAF or MEK inhibitor with an AKT or mTOR inhibitor. These combinations should be available for clinical testing in patients progressing on BRAF inhibitors.

## Introduction

BRAF^V600E^ is a dominant activating mutation in melanoma resulting in a constitutive activation of the mitogen-activated protein kinase (MAPK) pathway and uncontrolled cell growth [1], [2]. Its role as a driver mutation for this cancer is validated by the high rate of tumor responses in patients with BRAF^V600E^ mutant metastatic melanoma treated with the type I RAF inhibitor vemurafenib (previously know as PLX4032 or RG7204) [3]. These clinical results with vemurafenib highlight that, despite the presence of multiple other genomic alterations in advanced melanoma, metastatic lesions with a BRAF^V600E^ mutation have all the features of oncogene addiction [4]. However, it is likely that, after the initial tumor response, secondary alterations in melanoma cells may contribute to the development of acquired resistance to vemurafenib and other type I RAF inhibitors with specific antitumor activity against mutated BRAF, such as dabrafenib (previously GSK2118436) [5].

Similar to other cancers, melanomas have frequent alterations in the phosphatidylinositol 3-kinases (PI3K) and v-akt murine thymoma viral oncogene homolog 1 (AKT) pathway, another key signal transduction pathway governing cell growth and survival. The most common alterations are genomic or functional loss of PTEN and amplification and point mutations in AKT [2]. Multiple pathways are activated downstream of AKT, the major one going through the mammalian target of rapamycin (mTOR) and its downstream effector ribosomal protein S6 kinase, 70-KD, 1 (RPS6KB1 or herein as p70 S6K1). It has been postulated that cells with mutations in BRAF may require co-operating alterations in PTEN or AKT to activate both main signal transduction pathways [6]. This is opposed to melanomas with NRAS mutations, since RAS mutations can provide oncogenic signal through both the MAPK and the PI3K/AKT pathways. Therefore, approaches to simultaneously inhibit both the MAPK and PI3K/AKT pathways have been proposed in melanoma [7]. The advent of highly specific inhibitors for oncogenic BRAF with robust activity in BRAF^V600E^ mutant melanoma [3], [8], [9], [10] and the clinical development of specific inhibitors of PI3K, AKT and mTOR, provide the tools to translate these concepts into the clinic.

Analysis of clinical samples provided evidence that the antitumor activity of vemurafenib is mediated by inhibition of ERK signaling [8]. In addition, preclinical data had suggested that BRAF^V600E^ mutant melanomas may continue to depend on the MAPK even after progressing on BRAF inhibitors, through the reactivation of phosphorylated ERK in resistant cells [11], [12]. Since MEK1/2 is the required signaling node between RAF and ERK, it had been postulated that a maintained dependence on the MAPK pathway in RAF inhibitor-resistant cells could be treated with specific MEK inhibitors. Based on these observations, clinical trials are underway to block MEK in patients whose BRAF^V600E^ mutant melanoma had a response but then progressed on BRAF inhibitors like vemurafenib or dabrafenib.

In this study we first tested the concept of treating with a MEK inhibitor upon progression on a BRAF inhibitor in selected melanoma cell lines that encompassed cell lines with primarily resistance to vemurafenib, those with acquired resistance to vemurafenib after *in vitro* exposure, and those established from patient-derived biopsies progressing after vemurafenib. Recent studies have shown that in addition to the dependence on MAPK pathway, over expression of receptors such as the platelet-derived growth factor beta (PDGFRβ) or the insulin growth factor-1 receptor (IGF-1R), which are upstream of PI3K/AKT pathway, may play important roles in the resistance to BRAF inhibitors [13], [14]. Therefore, we also investigated the activity of the AKT pathway and its possible effect on the resistance of melanoma cells to inhibitors of MAPK pathway. We also examined whether the induction of AKT signaling by inhibitors of MAPK pathway may in part be caused by the activation of the feedback mechanisms originating from the downstream factors in AKT pathway. Our results suggest that most BRAF^V600E^ mutant melanomas not responding to vemurafenib are also cross-resistant to single agent MEK inhibitors, but co-targeting of the AKT/mTOR pathway provides means of treating most of these resistant cells.

## Materials and Methods

### Reagents and cell lines

Vemurafenib (also known as PLX4032, RG7204 or RO5185426) was obtained under a materials transfer agreement (MTA) with Plexxikon (Berkeley, CA) and Roche (Nutley, NJ). It was dissolved in DMSO (Fisher Scientific, Morristown, NJ) to a stock concentration of 100 mM. AZD6244 was purchased from Selleck Chemicals (Houston, TX) and dissolved in DMSO to a 100 mM stock. The isozyme-selective AKTi VIII (AKTi, Calbiochem, Cambridge, MA) was dissolved in DMSO to a stock concentration of 10 µM. Rapamycin in EtOH (Calbiochem) was used for the drug combination experiments at the noted concentrations. Human melanoma cell lines (M series) were established from patient's biopsies under UCLA IRB approval #02-08-067 as previously described [15]. Cells were cultured in RPMI 1640 with L-glutamine (Mediatech Inc., Manassas, VA) containing 10% (unless noted, all percentages represent volume to volume) fetal bovine serum (FBS, Omega Scientific, Tarzana, CA) and 1% penicillin, streptomycin and amphotericin B (Omega Scientific). All cell lines were mycoplasma free when periodically tested using the Mycoalert assay (Lonza, Rockland, ME).

### Establishment of vemurafenib-resistant cell lines derived from patient's tumor biopsies

Four cell lines were derived from patients participating in the phase I (NCT00405587) or phase II (NCT00949702) clinical trials with vemurafenib as previously described [13]. These cell lines were obtained after written informed consent under the UCLA Institutional Review Board approval IRB#02-08-067. This same IRB approval covered the establishment and use of other cell lines included in this research. M370, M376 and M395 were derived from tumor biopsies of metastatic melanoma lesions progressing after an initial objective response to vemurafenib treatment. M380 was derived from a melanoma metastasis primarily resistant to vemurafenib from the start of treatment with the drug. In brief, tumor biopsies were minced and enzymatically treated with 0.1% type I collagenase (Sigma Immuno Chemicals, Fluka Chemie, Buchs, Switzerland) and 0.02% DNase (Boehringer Mannheim, Mannheim, Germany) in complete tissue culture medium for 2 hours. After centrifugation, cells were resuspended in RPMI 1640 with L-glutamine (Mediatech Inc., Manassas, VA) containing 20% FBS, and 1% penicillin, streptomycin and amphotericin B, and incubated at 37°C in a 5% CO_2_- and water-saturated incubator. The medium was changed when most cells had attached and cell cultures were then propagated and passaged *in vitro* as needed. These *in vivo* resistant cultures were not routinely selected with constant exposure to vemurafenib.

### *In vitro* acquired vemurafenib resistance

To generate cell lines with *in vitro* acquired resistance, BRAF^V600E^ mutant melanoma cell lines with *in vitro* sensitivity to vemurafenib were plated in T25 flasks and treated with their respective IC_50_ of vemurafenib as described previously [13]. The vemurafenib concentration was then either increased or remained the same depending on the previous viability count, for 120-hour periods, until a subline grew progressively in the presence of vemurafenib above the IC_50_ for the parental cell line. The subline was labeled as the parental cell line followed by “AR” for acquired resistance.

### Cell proliferation and viability assays

Melanoma cell lines were treated in triplicate with vemurafenib, AZD6244, AKTi or rapamycin (or the combinations) and parallel vehicle control in the given concentrations for 72 hours. Cell viability was measured using a tetrazolium compound [3-(4,5-dimethylthiazol-2-yl)-5- (3-carboxymethoxyphenyl)-2-(4-sulfophenyl)-2H-tetrazolium (MTS)-based colorimetric cell proliferation assay (Promega, Madison, WI) as previously described [15]. Each experiment has been repeated at least twice and the most reproducible study is presented.

### Oncogene characterization of cell lines

Information for the oncogenic characterization of the cell lines tested herein was based mainly on data we have previously reported [13], [15]. The additional analysis of gene copy changes based on DNA extracted from melanoma cell lines hybridized onto HumanOmni1-Quad_v1-0_B (Illumina Inc., San Diego, CA).

### siRNA transfection

Cell lines were transfected with the gene-specific or no target control siRNAs (Dharmacon, Lafayette, CO). To perform the transfection, Lipofectamine™ RNAiMAX reagent (Invitrogen, Carlsbad, CA) was used according to the Reverse Transfection method described by the manufacturer. In brief, 40 pmol of each siRNA was diluted separately into 250 µl of serum free phenol red free RPMI 1640. To each diluted siRNA, 3 µl of Lipofectamine™ RNAiMAX was added. Each transfecting mixture was added to 1.8×10^5^ cells suspended in 1.25 ml of the culturing media. Then, each sample was plated for protein isolation after 72 hours or RNA isolation after 48 hours or for the drug treatment after 24 hours and assessment of proliferation rate after 120 hours.

### Western blotting

Western blotting was performed as previously described [16]. Primary antibodies included p-AKT Ser473 and Thr308, AKT, p-S6K1 Thr389, S6K1, p-S6 Ser235/236, p-4EBP-1, S6, p-ERK Thr204/205, ERK, pMEK Ser217/221, MEK, 4EBP-1, cleaved caspase-3 and beta-actin (all from Cell Signaling Technology, Danvers, MA). The immunoreactivity was visualized by use of an ECL-Plus kit (Amersham Biosciences Co, Piscataway, NJ) and scanning of the blots by the Typhoon scanner (Amersham Biosciences Co, Piscataway, NJ).

### Apoptosis assay

To perform the apoptosis assay cells were treated with the solvent (DMSO) or 2 µM of vemurafenib, AZD6244, AKTi or 10 nM of rapamycin or combination of these compounds. After 48 hours of treatment, protein lysated were prepared from each condition and analyzed by Western blotting method for the detection of cleaved caspase-3.

#### RT-PCR for detection of S6K2 mRNA level

RT-PCR assay was used to investigate the efficiency of ribosomal protein S6 kinase, 2 (RPS6KB2 or here in S6K2) knockdown by siRNA cocktail at mRNA level. M238 and M238AR2 cell lines were transfected with a combination of 25 pmol of S6K2 siRNA plus 25 pmol of S6K1 siRNA or with 50 pmol of control SiRNA (as described in the above). Cells were harvested after 48 hours and total RNA was isolated by using RiboPure™ Kit (Ambion, Austin, TX). RT-PCR assays were performed on 350 ng of total RNA isolated from each sample by using SuperScript III One-Step RT-PCR System with Platinum® Taq kit (Invitrogen, Carlsbad, CA). Specific S6K2 set of primers (Forward 5′CTGAGCGGAACATTCTAGAGT 3′ and Reverse 5′-AAGTCGGTCAGTTTGATGTGG-3′) were used to amplify a cDNA fragment of ∼300 base pares. The primers span the sides of an intron and do not amplify the genomic DNA. As a control for the integrity and level of RNA, GAPDH was amplified by specific Forward (5′-GTCAACGGATTTGGTCGTATT 3′) and Reverse (5′-AGTCTTCTGGGTGGCAGTGAT 3′) primer set. RT-PCR cycles were as follows: Reverse transcription at 50°C for 30 minute, 95°C for 2 minutes, 27 cycles of 94°C (15 Sec), 56.5°C (30 Sec), 68°C (30 Sec), and 68°C for 5 minutes. The samples were analyzed on 1.5% agarose gel.

### Statistical analysis

To determine synergistic, additive, or antagonistic effects of the drug combinations, we used the combination index method of Chou and Talalay [17] using the CalcuSyn software (version 2.0 Biosoft, Cambridge, UK). This method takes into account both potency [median dose (Dm) or IC_50_] and the shape of the dose-effect curve (the *m* value) to calculate the combination index (CI). A CI equal to 1 indicates an additive effect; a CI less than 1 indicates synergy. With the use of CalcuSyn software, synergy is further refined as synergism (CI = 0.3–0.7), strong synergism (CI = 0.1–0.3), and very strong synergism (CI<0.1).

## Results

### Frequent cross-resistance to vemurafenib and AZD6244

**Figure 1:**
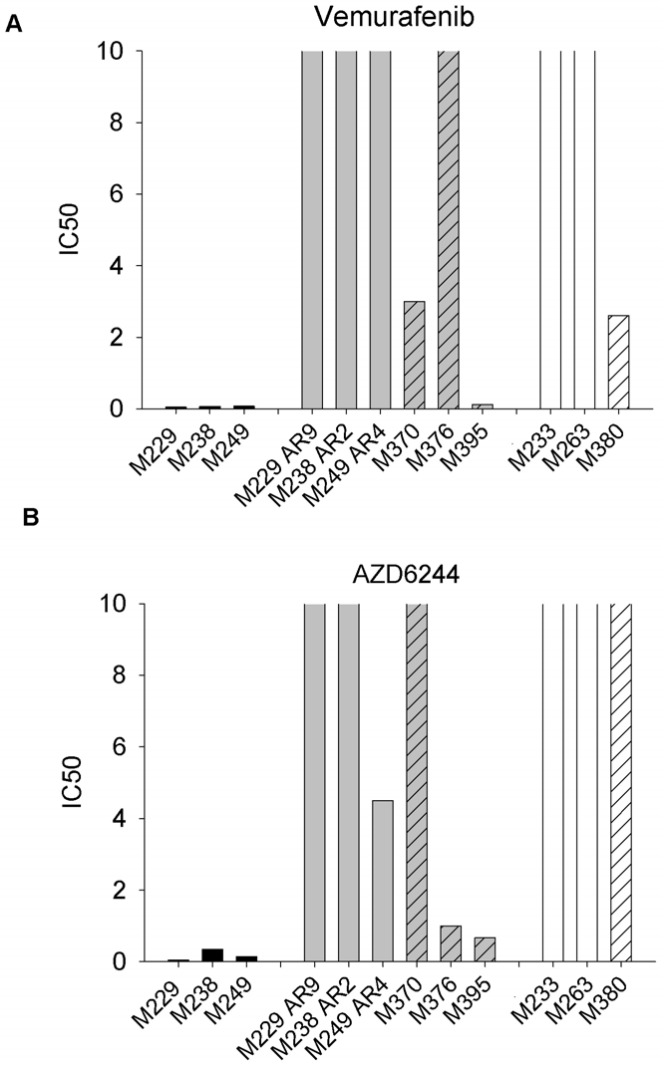
IC_50_ values of BRAF^V600E^ mutated melanoma cells after exposure to vemurafenib (a) or AZD6244 (b). The cells were treated for 120 hours (vemurafenib) or 72 hours (AZD6244). Cell viability was determined by MTS colorimetric assay. IC_50_ values (x-axis) are expressed in µM for vemurafenib or AZD6244. Black columns: Parental cell lines sensitive to vemurafenib. Gray columns: Sublines with *in vitro* acquired resistance to vemurafenib. Gray columns filled with coarse striped pattern: Cell lines derived from progressive lesions in patients treated with vemurafenib. White columns: vemurafenib primarily resistant cell lines. White column filled with coarse striped pattern: Cell line derived at baseline from a patient who did not respond clinically to vemurafenib.

We analyzed if melanoma cell lines resistant to vemurafenib would be sensitive to the MEK inhibitor AZD6244 using MTS assays (Figure 1 and Figure S1). Cell lines with IC_50 s_ above 10 µM for each agent were considered resistant. M229, M238 and M249 are three BRAF^V600E^ mutant parental cell lines sensitive to vemurafenib [15]. These three cell lines also demonstrated high sensitivity to AZD6244. The corresponding sublines with acquired resistance after continuous *in vitro* culture in increasing concentrations of vemurafenib also exhibited a complete (M229-AR9, M238-AR2) or a partial (M249-AR4) resistance to single agent AZD6244. The other three cell lines (M370, M376, M395) obtained from metastatic melanoma lesions surgically resected from patients progressing on vemurafenib had different sensitivities to both agents. M370 was established from an intra-cardiac mass which developed six months after starting on vemurafenib. It corresponds to the sample labeled as Pt48 R in ref. [13]. This cell line maintained partial *in vitro* sensitivity to vemurafenib but was resistant to AZD6244. M376 was established from a lymph node metastatic lesion that partially regressed on vemurafenib but increased in size 10 months after starting the therapy. It corresponds to the sample labeled as Pt55 R in ref. [13]. This cell line was markedly resistant to vemurafenib but was sensitive to AZD6244. M395 was established from an adrenal metastasis that had partially regressed on vemurafenib therapy during 5 months, but then slowly increased in size. Surprisingly, this cell line maintained *in vitro* sensitivity to both vemurafenib and AZD6244. For these studies, the cell lines obtained from patients treated with vemurafenib had been cultured *ex vivo* without the presence of low dose vemurafenib for several passages, which is different from studies performed by Nazarian *et al.* [13]. M233 and M263 are previously established BRAF^V600E^ mutant cell lines with primary resistance to vemurafenib [15]. They also demonstrated primary resistance to AZD6244. M380 is a cell line established from a baseline subcutaneous metastasis in a patient with metastatic melanoma who had progression with vemurafenib treatment at restaging scans 6 weeks after the initiation of treatment with vemurafenib. The biopsied lesion transiently decreased in size for two weeks while on vemurafenib but it then rapidly progressed. This cell line was partially sensitive to vemurafenib *ex vivo* but completely resistant to AZD6244. Thus, with the exception of M249-AR4 and M376, BRAF^V600E^ mutant melanoma cell lines resistant to vemurafenib have cross resistance to the MEK inhibitor AZD6244.

### Acquired resistance to vemurafenib mediated by a secondary NRAS mutation leads to sensitivity to a MEK inhibitor

**Figure 2:**
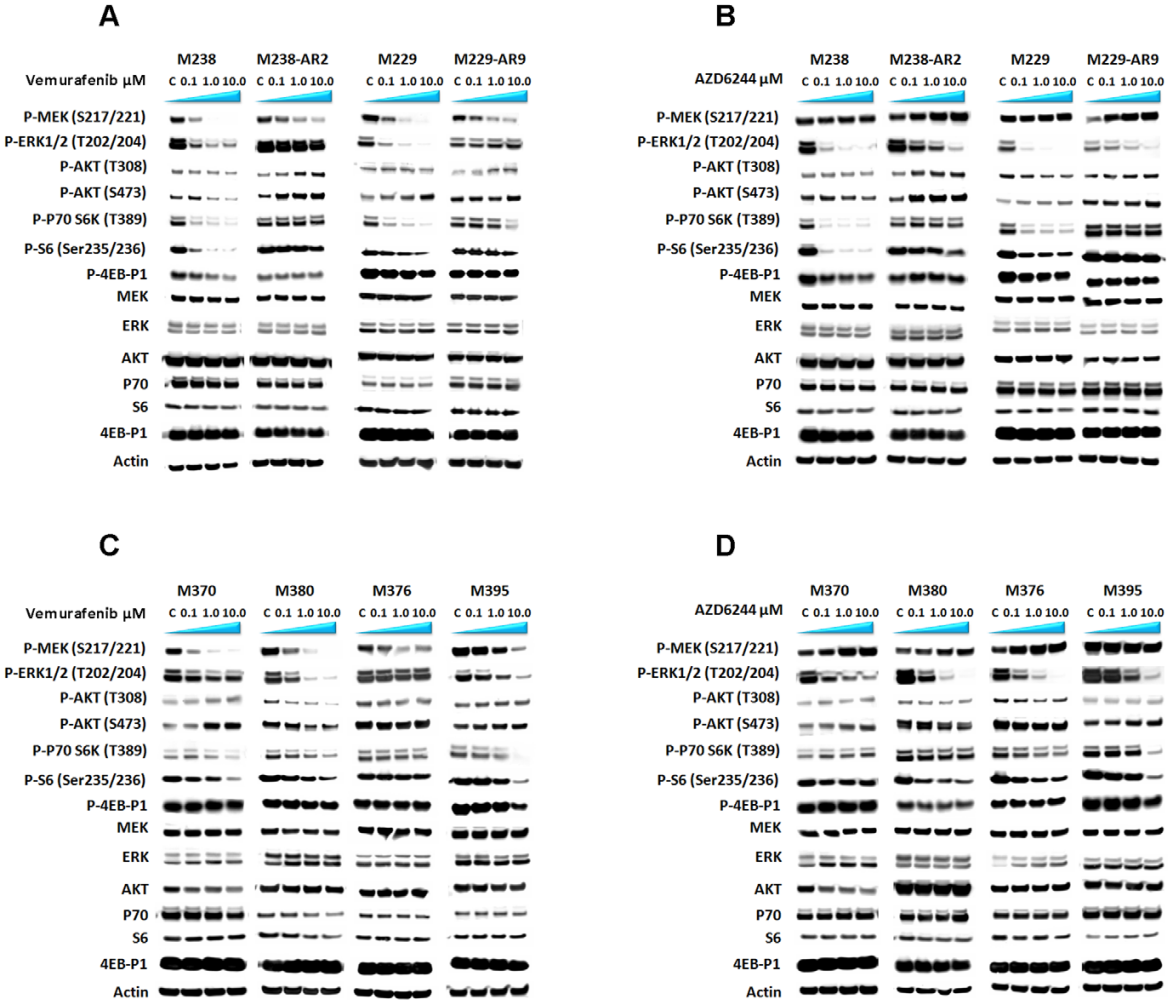
Effects of vemurafenib or AZD6244 on MAPK and PI3K/AKT pathways in BRAF^V600E^ mutated cell lines. Western blot analysis of phosphorylated and the total amount of key proteins in the MAPK and PI3K/AKT pathways after 24 hours of exposure to the solvent (DMSO), or various concentrations of the BRAF inhibitor vemurafenib or the MEK inhibitor AZD6244. The vemurafenib-sensitive M238 and M229 cell lines and the vemurafenib *in vitro* acquired resistant sublines M238-AR2 and M229-AR9 were cultured at different concentrations of vemurafenib (a) or AZD6244 (b). The vemurafenib-resistant cell lines derived from patient's tumor biopsies M370, M376, M395 and M380 were cultured in different concentrations of vemurafenib (c) or AZD6244 (d). p70 and p-p70 S6K in this figure are referred to S6K1 and phosphorylated form of S6K1, respectively.

We explored the oncogenic alterations in this panel of cell lines using targeted oncogene sequencing and SNP arrays (Table 1 and references [13], [15]). The presence of a secondary mutation in NRAS^Q61K^, in addition to the BRAF^V600E^ mutation, in the *in vitro* acquired resistant cell line M249-AR4 and in the patient-derived acquired resistant cell line M376, was associated with resistance to vemurafenib but sensitivity to AZD6244, corroborating the earlier study [13]. Upon treatment of M376 with vemurafenib (24 hours), despite a partial decrease in p-MEK, no obvious decrease in p-ERK1/2 or in factors downstream of AKT was observed. On the contrary, AZD6244 caused the accumulation of p-MEK, as well as decreases in p-ERK1/2 and p-S6 (Figure 2). These findings indicate that vemurafenib fails to inhibit the NRAS^Q61K^-induced signaling while the MEK inhibitor AZD6244 blocks the pathway, and consequently inhibits the cell proliferation in these cell lines. Secondary mutations in BRAF, KRAS, NRAS or HRAS were not noted in any other cell line [13]. These data provide strong indication that in BRAF mutant cells that have a secondary NRAS mutation, or perhaps other mechanisms of acquired resistance that reactivate MEK, addition of a MEK inhibitor can result in secondary responses. Therefore, we focused mostly on the non-NRAS secondary mutation cells since it was not clear what signaling pathway should be blocked to control them. We continued by studying MAPK and alternative signaling through the PI3K/AKT pathway on non-NRAS secondary mutated resistant cell lines.

**Table 1:** Cell line characterization.

Cell Line	Vemurafinib sensitive/resistance origin	Main oncogenic events
M229	*In vitro* naturally sensitive	*BRAF^V600E^* homozygous
		*AKT1* amplification
		*PTEN* heterozygous deletion
M229-AR9	*In vitro* acquired resistance	*BRAF^V600E^* homozygous
		*AKT1* amplification
		*PTEN* heterozygous deletion
M238	*In vitro* naturally sensitive	*BRAF^V600E^* heterozygous
		*PTEN* heterozygous deletion
		*CDKN2A* homozygous deletion
M238-AR2	*In vitro* acquired resistance	*BRAF^V600E^* heterozygous
		*PTEN* heterozygous deletion
		*CDKN2A* homozygous deletion
M249	*In vitro* naturally sensitive	*BRAF^V600E^* heterozygous
		*PTEN* homozygous deletion
M249-AR4	*In vitro* acquired resistance	*BRAF^V600E^* heterozygous
		*NRAS^Q61K^* heterozygous
		*PTEN* homozygous deletion
M370	Patient-derived from a cardiac mass with acquired resistance	*BRAF^V600E^* heterozygous
M376	Patient-derived from a nodal metastasis with acquired resistance	*BRAF^V600E^ heterozygous*
		*NRAS^Q61K^ heterozygous*
		*PTEN heterozygous deletion*
M380	Patient-derived from a subcutaneous mass with natural resistance	*BRAF^V600E^ heterozygous*
		*CDKN2A homozygous deletion*
M395	Patient-derived from an adrenal mass with acquired resistance	*BRAF^V600E^ homozygous*
		*CDKN2A homozygous deletion*
M233	*In vitro* naturally resistant	*BRAF^V600E^* heterozygous
		*AKT1* amplification
		*PTEN* homozygous deletion
M244	*In vitro* naturally resistant	*NRAS^Q61K^ heterozygous*
M263	*In vitro* naturally resistant	*BRAF^V600E^* heterozygous

### Drug induced alterations of MAPK signaling and differential modulation of the AKT pathway in vemurafenib-sensitive and -resistant cell lines

We selected two pairs of parental and their *in vitro* acquired resistant sublines and four of the patient-derived cell lines to explore signaling pathways that may be differentially modulated upon treatment with the RAF or MEK inhibitors for 24 hours (Figure 2). Vemurafinib treatment of parental cell lines caused a decrease in the level of p-MEK in a dose dependent manner. However, this decrease was less extensive in the highly resistant cell lines (M238-AR2 and M229-AR9). Similarly, in M238-AR2 and M229-AR9, vemurafenib was inefficient in causing a decrease in the p-ERK1/2 levels when analyzed at the 24 hour time point (note that the timing of these studies analyzing p-ERK is later than the evidence of maintained ability to inhibit p-ERK at earlier time points in our prior studies [13], suggesting p-ERK recovery after the initial suppression). On the contrary, AZD6244 treatment induced higher levels of p-MEK in the cell lines that showed *in vitro* resistance to vemurafenib (M229-AR9, M238-AR2, M370, and M380), as well as in M376 which was sensitive. p-ERK1/2 levels were lower in all the AZD6244 treated samples regardless of their sensitivity to either one of the drugs.

The differences in p-AKT/p-p70 S6K1/p-S6 pathways were more pronounced between sensitive and resistant cell lines. Vemurafinib and AZD6244 induced similar changes in p-AKT levels. However, it seemed that these changes were cell line-dependent, showing simultaneous increases in p-AKT T308 and S473 (suggesting feedback) in the resistant cell lines M238-AR2, M229-AR9 and M370, while there were no obvious changes in M395, and decreases in both p-AKT levels in M238 and M380. Thus, none of the cell lines highly resistant to vemurafenib showed decreases in one or both p-AKT levels after the exposure to the drugs. In fact, vemurafenib (and to a lesser extent AZD6244) treatment of vemurafenib-acquired resistant cell lines consistently increased p-AKT levels, with the notable exception of the N-RAS mutated line, M376. Exposure of sensitive cell lines, M229 and M238, to even low concentrations of the drugs caused drastic decreases in their p-p70 S6K1 and p-S6 levels. However, particularly at lower concentrations of the drugs, there was less or no effect on p-p70 S6K1 levels in the *in vitro* acquired resistant cells M238-AR2 and M229-AR9, and the patient-derived resistant cell lines. Both vemurafenib and AZD6244 caused decreases in p-S6 levels in sensitive cell lines but almost no clear changes in the their resistant sublines. However, it seemed that the decrease in p-S6 was not associated with the pattern of response to the drugs except when accompanied by a decrease in p-p70 S6K1. 4-EBP-1 is another important factor downstream of AKT/mTOR pathway that has been reported to link this pathway to the MAPK pathway [18]. Only the sensitive cell lines M238 and M229 showed detectable deceases in levels of p-4-EBP-1 after exposure to vemurafenib or AZD6244. Altogether, these data suggest that in cell lines with resistance to vemurafenib, regardless of the presence or absence of a secondary NRAS mutation, this agent at least partially maintained the ability to inhibit phosphorylation of MEK. The unexpected effect of MAPK (in particular BRAF) inhibition on p-AKT increase in non-NRAS mutated, vemurafenib-acquired resistant lines suggested crosstalk between AKT and the MAPK pathway and points to potential therapeutic opportunities in the AKT-mTOR pathway.

### Reversal of the pattern of resistance with genetic silencing of RICTOR or S6K1 and 2

**Figure 3:**
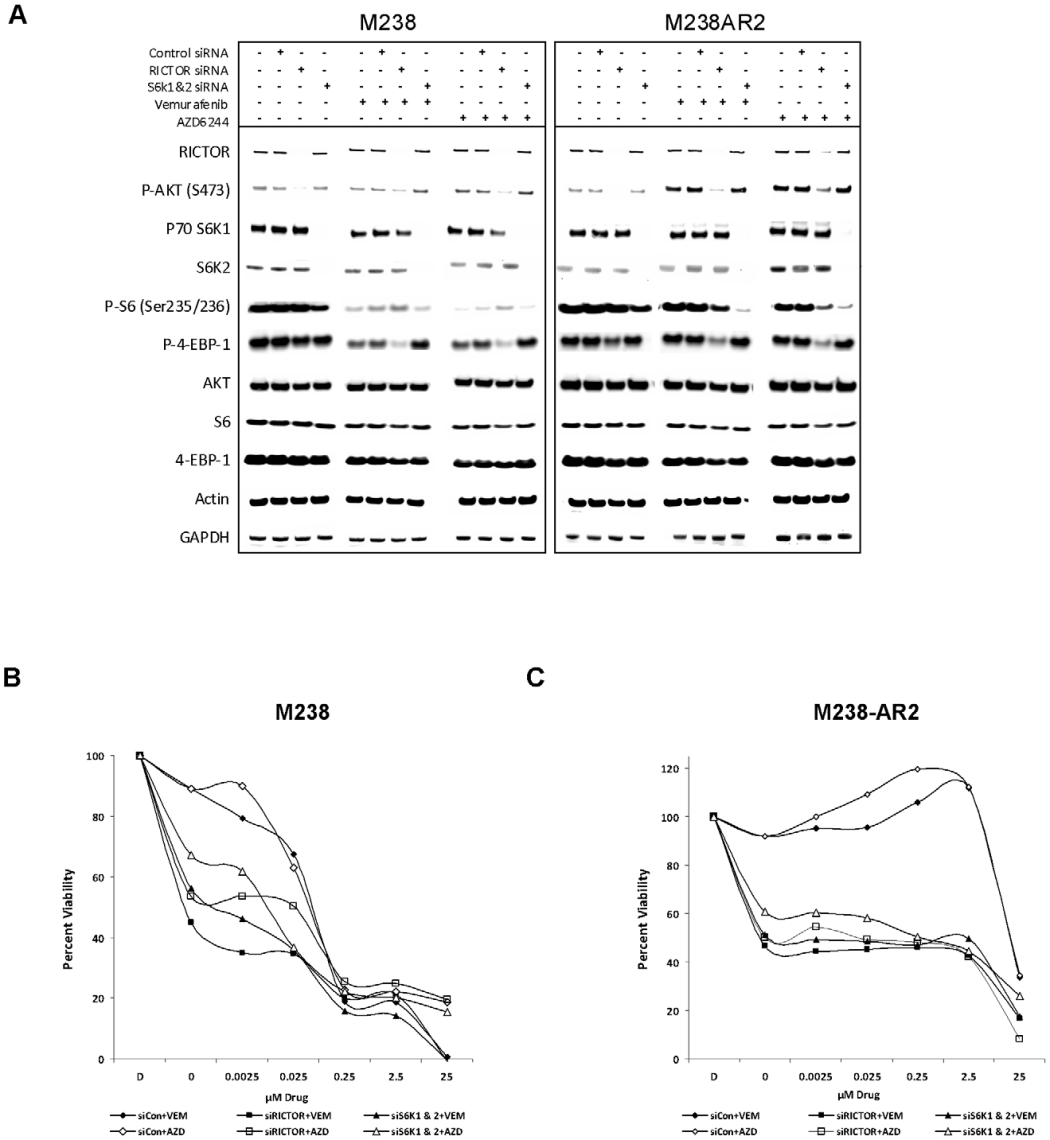
Effects of both S6K1 and S6K2 or RICTOR siRNA knockdown combined with vemurafenib or AZD6244. The efficiency of siRNA knockdowns and their effects on downstream signaling determined by Western blot analysis of protein lysates or in the cases of S6K2 and GAPDH by RT-PCR of isolated mRNA (a). M238 parental (b) and M238-AR2 resistant subline (c) were transfected with siRNAs for either RICTOR or combined S6K1 & 2 or non target control siRNAs and cultured in increasing concentrations of vemurafenib or AZD6244. The effects of knockdowns on resistance and growth inhibition were analyzed after 120 hours by an MTS assay. D in each graph refers to the un-transfected untreated cells and is used as the 100% reference point for all the conditions in each graph.

mTORC complexes have been described as the central node for regulation of growth and metabolism. RICTOR is one of the proteins in mTORC2 complex that phosphorylates AKT at Ser473 through a feedback mechanism. This phosphorylation is necessary for the complete activation of AKT. p70 S6K1 is one of the key factors downstream of mTOR that phosphorylate and induces S6 activity. Given the pattern of p-AKT induction primarily in the non-NRAS mutant acquired resistant melanoma cell lines we focused on the effects of inhibiting the mTORC complexes or p70 S6K1. In M238-AR2 and its parental cell line, transient knockdown of each target was achieved by transfection of cells with the target specific siRNAs pool that resulted in a reproducible knockdown of at least 70% at protein levels (Figure 3a). A highly homologous gene to p70 S6K1 is S6K2 which has been suggested to compensate for the lack of p70 S6K1 function in animal knockout studies [19]. Therefore, in order to avoid the redundant functional effect of S6K2 in the context of p70 S6K1 knockdown, pooled siRNAs for the knockdown of S6K2 was mixed with those for the knockdown of S6K1. The knockdown of S6K2 was detected by RT-PCR using trans-intron specific primers for this gene (Figure 3a). The knockdown of S6K1 and 2 caused a decrease in phosphorylation of p-S6 in all cases. Similar to previous experiments, in M238 parental cell line treatment with vemurafenib or AZD6244 alone was sufficient to decrease the p-S6 levels visibly. Interestingly, in M238-AR2, such a decrease in p-S6 level could only be achieved if the knockdown of S6K1 and 2 was combined with either vemurafenib or AZD6244 treatment (Figure 3a). These results suggest that in this resistant cells, S6 is a cross-talk point between MAPK and AKT pathways and therefore inhibition of both pathways is required to down-regulate the activity of this protein. In the growth assays, genetic silencing of both S6K1 and S6K2 caused a significant decrease in the growth rates of the cells and also reversed the growth inducing effect of both drugs which is observed in the resistant cell line M238-AR2 (Figure 3b and 3c).

As it was expected, knockdown of RICTOR caused an evident decrease in phosphorylation of p-AKT at Ser473. Interestingly, knockdown of RICTOR also caused a clear decrease in phosphorylation of 4-EBP-1 particularly in the presence of vemurafenib or AZD6244, and also a detectable decrease in p-S6 level only in M238-AR2 treated with the agents (Figure 3a). These results suggest that in melanoma cells resistant to inhibitors of MAPK pathway, 4-EBP-1 is a cross-talk point between MAPK and AKT pathways. Therefore blocking of both pathways is necessary to down regulate activities of proteins downstream of AKT pathway. In growth inhibition assays, genetic silencing of RICTOR significantly decreased the growth rates and reversed the growth inducing effect of vemurafenib and AZD6244 in the resistant cell line (Figure 3b and 3c). These findings suggest that the mTORC2 feedback that phosphorylates AKT may play an important role in maintenance or induction of cell growth and therefore causing the resistance to MAPK pathway inhibitors.

RAPTOR is one of the main proteins in mTORC1 complex. In our experiments, knockdown of RAPTOR caused detectable decreases in phosphorylation of p-p70 S6K1 in both parental and resistant cell line. However, knockdown of RAPTOR in these cell lines caused no detectable decrease in p-S6 level (Figure S2a). Knockdown of RAPTOR decreased the growth rate of both cell lines. However, it could not prevent the growth-inducing effect of vemurafenib and AZD6244 in the M238-AR2 resistant cell line, which can be observed even at up to 1 µM level of these drugs (Figure S2b). This phenomena in our RAPTOR knockdown cells can be due to the lack of decrease in phosphorylation of S6 in combination with the described feedback mechanism that is initiated by the mTORC1 inhibition and causing over activity of mTORC2 and consequently inducing higher phosphorylation of AKT Ser473 [20].

### Frequent reversal of resistance to vemurafenib or AZD6244 with concomitant inhibition of AKT or mTOR

**Figure 4:**
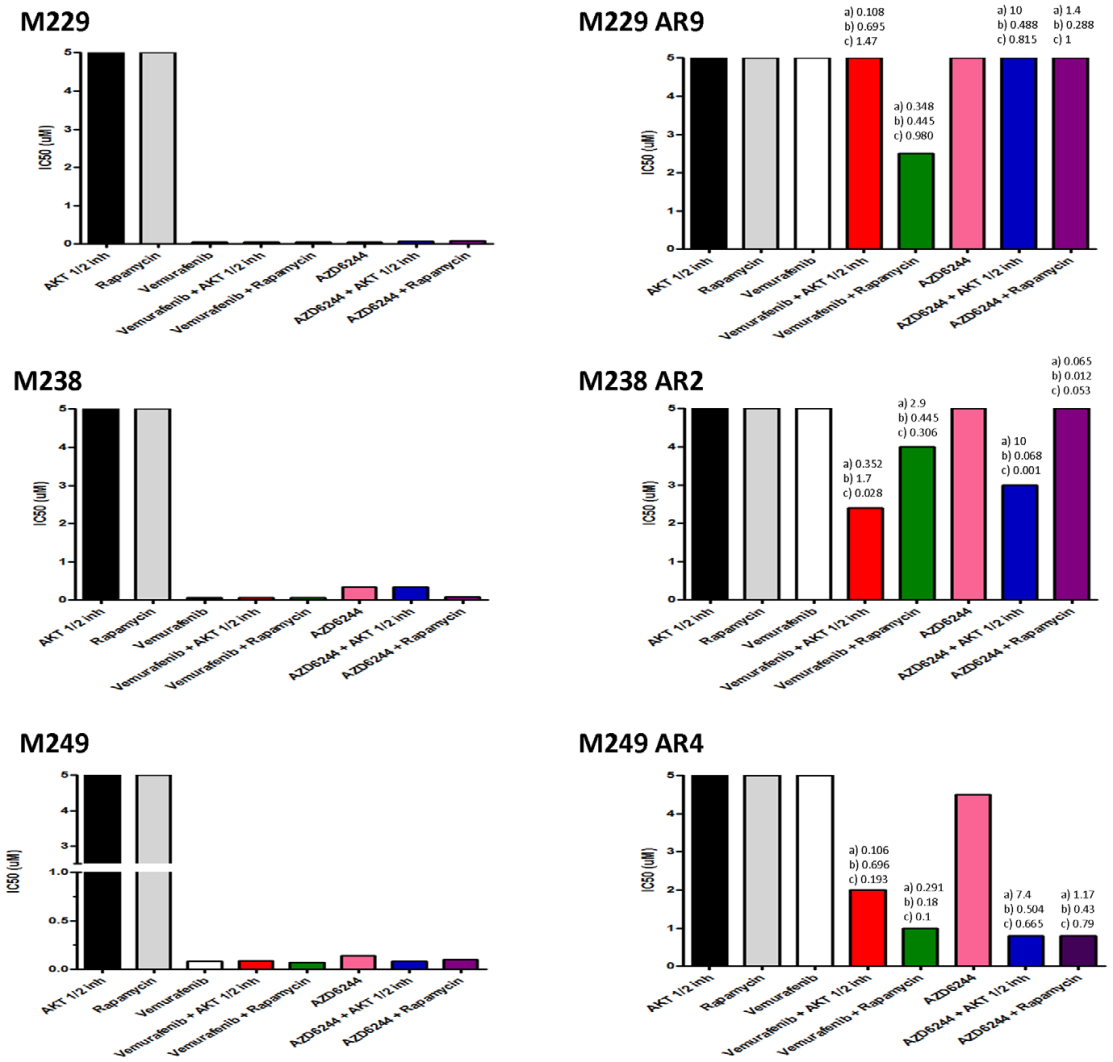
AKTi or rapamycin combined with vemurafenib or AZD6244 in vemurafenib-sensitive and -acquired resistant cell lines. IC_50_ of the parental cell lines M229, M238 and M249, and the acquired resistance sublines M229-AR9, M238-AR2 and M249-AR4 determined in an MTS assay using single agent AKTi, rapamycin, vemurafenib or AZD6244, or in combinations. Vemurafinib or AZD6244 in combination with AKTi were tested at 1∶1 ratios at concentrations of 0.1, 1 or 5 µM, or with rapamycin at 0.1, 1 and 5 nM. For the combination studies the IC_50_ bar represents either vemurafenib or AZD6244 used in the combination. The combination indexes (CI) were calculated by the Chou-Talalal method and denoted over each column where a synergistic (CI<1) effect was noted. There are three CIs per condition reflective of the three different concentrations tested, 0.1; 1 and 5 for each drug at 1∶1 ratio (µM for PLX; AZD and AKTi; nM for rapamycin).

**Figure 5:**
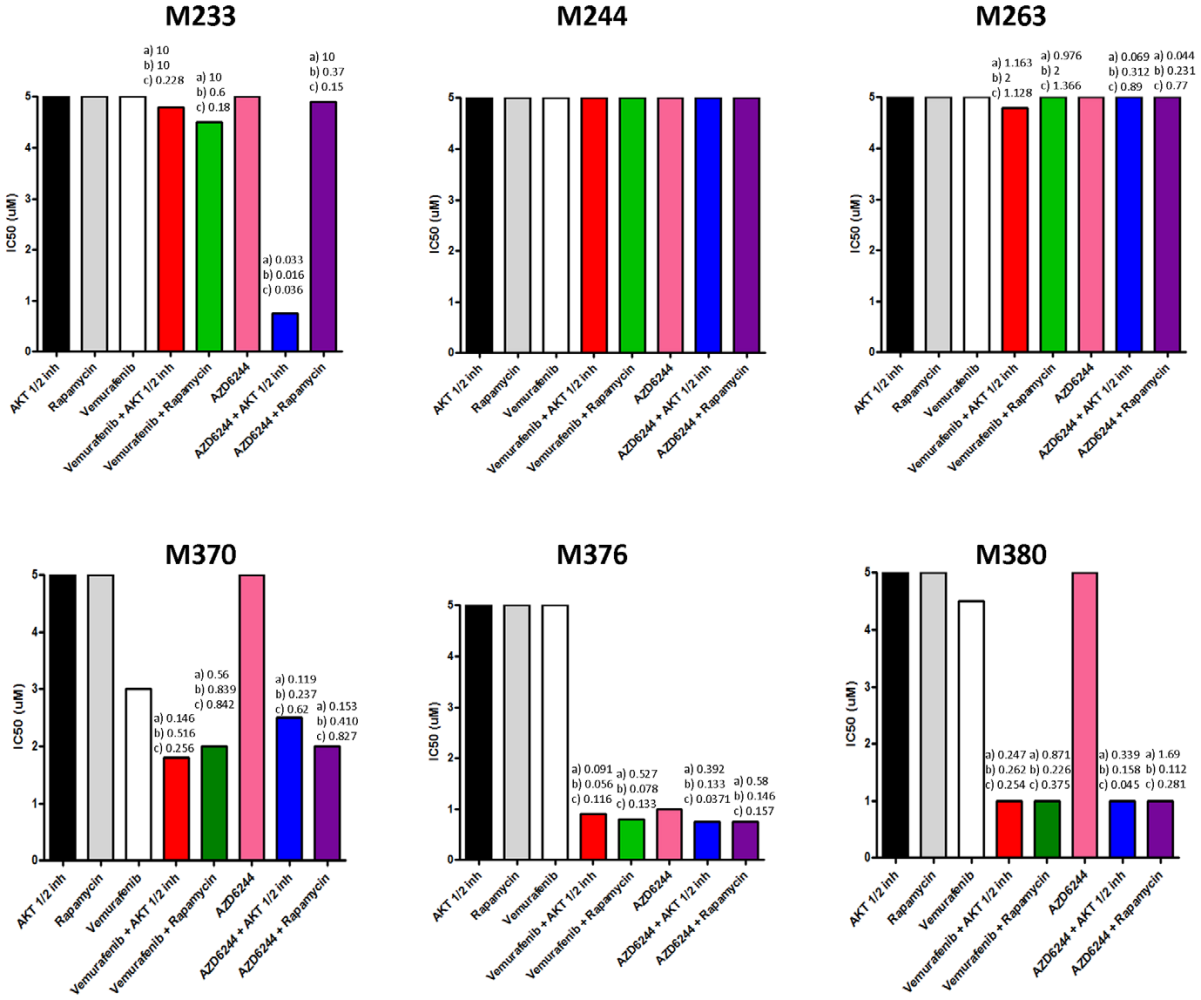
AKTi or rapamycin combined with vemurafenib or AZD6244 in patient-derived vemurafenib-primary/-acquired resistant cell lines. IC_50_ of the primarily resistant cell lines M233, M244 and M263, and the patient-derived acquired resistance cell lines M370, M376 and the primarily resistant patient-derived cell line M380 determined by an MTS assay and analyzed for synergistic effects as described in Figure 4.

To examine clinically-relevant means of addressing primary or acquired resistance to single agent MAPK inhibitors we tested the addition of an AKT1/2 or a mTORC1 pharmacological inhibitor. All cell lines were resistant to single agent AKT inhibitor (AKTi) or rapamycin (Figures 4 and 5). Among the parental and acquired resistance subline pairs, resistance to vemurafenib in M229-AR9 was partially reversed with the addition of rapamycin but not with the AKTi, and resistance to AZD6244 could not be reversed with either agent (Figure 4). In M238-AR2, resistance to vemurafenib and AZD6244 was better reversed with the AKTi compared to rapamycin which also recapitulates our results on effects of RICTOR versus RAPTOR knockdowns on resistance (Figure 3c and figure S2c). For M249-AR4 both AKTi and rapamycin provided strong synergistic effects with either vemurafenib or AZD6244. The primarily resistant established cell lines continued to display cross-resistance to vemurafenib and AZD6244 in most cases, except for the addition of AKTi to AZD6244 (but not vemurafenib) in M233 (Figure 5). Among the patient-derived cell lines there was evidence of high synergy with the addition of the AKTi or rapamycin to either vemurafenib or AZD6244 in all instances. This includes M376, which is highly sensitive to single agent AZD6244, and the addition of AKTi or rapamycin resulted in further synergistic inhibitory effects.

To investigate the effect of each one of these drugs and their combinations in induction of apoptosis in *in vitro* sensitive/adaptive resistant pair cell lines, we detected cleaved caspase-3 (CC3) levels by Western blotting method (Figure S3). In all three parental sensitive cell lines (M229, M238, M249) significant amount of CC3 was detected after 48 hours of treatment with vemurafenib, AZD6244 or their combinations with rapamycin and AKTi. As it was expected, M249-AR4 with a secondary mutation in NRAS showed no detectable CC3 after treatment with vemurafenib, rapamycin, AKTi and their combinations. However, AZD6244 and its combination with rapamycin and AKTi induced noticeable levels of CC3. In the cases of M238-AR2 and M229-AR9, only low levels of CC3 was detectable after treatment with AZD6244 and its combination with rapamycin and AKTi.

## Discussion

The work presented herein provides evidence of frequent cross-resistance to the BRAF inhibitor vemurafenib and the MEK inhibitor AZD6244 in cell lines with primary or acquired resistance to vemurafenib, with frequent reversal of the acquired resistance by the addition of inhibitors of the AKT/mTOR pathway. In this study only cell lines with a secondary NRAS mutation, that are sensitive to MEK inhibitor, were the exceptions to the cross-resistance to BRAF and MEK inhibitors. The cross-resistance between the BRAF and MEK inhibitors is rather surprising given the exquisite dependence that BRAF^V600E^ mutant melanomas have demonstrated on the MAPK pathway. In paired biopsies of patients treated with vemurafenib this agent demonstrated a dose-dependent inhibition of p-ERK, suggesting that the activity of this agent as inhibitor of oncogenic BRAF relies on efficient inhibition of MAPK pathway signaling [8]. The development of *in vitro* acquired resistance to PLX4720, an analogue of vemurafenib, has been linked to the re-activation of p-ERK [12]. In addition, acquired resistance to a different BRAF inhibitor, AZ628, was associated with alternate signaling from BRAF to CRAF again resulting in the re-activation of p-ERK [11]. Combined, these data had suggested that further inhibition of the MAPK pathway with a MEK inhibitor may be a way to treat acquired resistance to the BRAF inhibitor. In fact, this concept has been taken into the clinic with ongoing clinical trials, but our data predicts that sequential single agent treatment with a MEK inhibitor after developing acquired resistance to a BRAF inhibitor will only work (partially) in a subset of cases with secondary NRAS mutations.

As the molecular mechanisms of primary and acquired resistance to vemurafenib are being studied [13], [14], [21], [22] it will be important to tailor the treatments to be added or sequentially tested in patients progressing on this therapy. It is becoming clear that resistance to BRAF inhibitors will not follow the pathway of resistance of chronic myelogenous leukemia (CML) to imatinib, where secondary mutations in the *abl* kinase are the main mechanism of resistance [23]. The study of resistance mechanisms in the sublines with *in vitro* acquired resistance to vemurafenib and patient-derived resistant cell lines included in this and other studies suggest three main mechanisms of acquired resistance, the upregulation of the receptor tyrosine kinases such as PDGFR1β [13] or IGF1R [14], increased expression of the cancer Osaka thyroid (COT, also known as MAP3K8) kinase [21], or secondary mutations in NRAS [13] or MEK [22]. Secondary mutation in NRAS or MEK, or upregulation of COT suggests acquired resistance mechanisms that maintain dependence on the MAPK pathway. In our studies, two vemurafenib-resistant cell lines with an acquired NRAS^Q61k^ mutation secondary to their pre-existing BRAF^V600E^ mutation exhibited some sensitivity to a sequential treatment with a MEK inhibitor. Interestingly, these two cell lines with the secondary NRAS mutation also showed sensitivity to the combinations of drugs inhibiting both AKT and MAPK pathways. This may be due to a possible cross talk between mutated NRAS and AKT pathway. Possibility of such a cross talk holds clinical and scientific importance and would be interesting to be investigated in the future studies. Meanwhile, all other cell lines displayed resistance to the sequential treatment with the MEK inhibitor if they were resistant to vemurafenib. In this group of resistant cell lines, most have the PDGFRβ-mediated mechanism of acquired resistance [13]. This information suggests that the elucidation of the specific mechanisms of resistance to vemurafenib points out to different therapies to be added or used sequentially with BRAF inhibitors.

A recent study on resistance to an analog of vemurafinib, PLX4720, suggested that only in cell lines with PTEN deletion p-AKT is induced by this BRAF inhibitor, and lack of PTEN may play a role in preventing apoptosis of melanoma cell treated with this compound [24]. However, in our study we found that the PTEN null cell line M249 was very sensitive to both vemurafenib and AZD6244, which may be an outlier compared to prior reported data [24]. Interestingly, by continuous exposure of this BRAF^V600E^ mutant/PTEN null cell line to vemurafenib an acquired resistant cell line that was mediated by a secondary mutation in NRAS causing the resistance through the reactivation of the MAPK pathway. Moreover, our results from other *in vitro* acquired resistant cell lines indicated that regardless of the PTEN status, p-AKT could be induced by vemurafenib or AZD6244 treatment. These findings indicate that alterations in both MAPK and AKT pathways can be the cause of resistance to vemurafenib and induction of p-AKT in resistant melanoma cell lines is rather a more general phenomenon and not solely limited to PTEN mutant cell lines.

There is clear evidence of multiple levels of cross-talk between MAPK and PI3K/AKT pathways, and it has been shown that ERK can be phosphorylated by the AKT pathway (Figure S4) [19], [25]. Therefore, it is likely that treatments to inhibit alternative survival signaling in melanoma cells resistant to MAPK inhibitors will require co-inhibition of the PI3K/AKT pathway. The concept of simultaneous inhibition of the MAPK and the PI3K/AKT/mTOR pathways has been widely considered to treat altered oncogenic signals [7], [18], [20], [26], and at least one clinical trial combining a MEK inhibitor with an AKT inhibitor is currently underway (NCT01021748). Given the frequent cross-talk and feedback regulation between both pathways we explored the effects of vemurafenib or AZD6244 on p-AKT and its downstream factors as the key signaling molecules in cells with primary or acquired resistance to vemurafenib. Our approach was also based on the evidence that cells with resistance to PLX4720, an analogue of vemurafenib, have a MEK-independent survival drive that can be blocked by inhibitors of the PI3K/AKT/mTOR pathway [25]. In addition, in cell lines with acquired resistance to BRAF inhibitors through upregulation of IGF1R, resistance can be inhibited by the co-administration of a combination of a MEK and a PI3K inhibitor [14]. Indeed our experiments demonstrated a differential effect on the AKT/mTOR/S6K pathway in vemurafenib-sensitive and -resistant cells both when exposed to vemurafenib or AZD6244. The most profound effect was the persistence of p-p70 S6K1 in cross-resistant cell lines treated with either drug, but it was particularly more evident with the MEK inhibitor AZD6244. Genetic inhibition of both p70 S6K1 and S6K2 with siRNAs showed additive effects with either of the drugs to further decrease the phosphorylation of the downstream protein S6. It should be noted that S6 can be phosphorylated at Ser235 by p-ERK as well. Therefore, changes in phophorylation of S6 can be the result of alterations in activity of p-ERK or p-P70 S6K1 or both, and that can be the reason for the lack of direct correlation between phosphorylation of S6K and S6 in our pharmacological inhibition studies.

In this study, siRNA knockdown of RICTOR decreased p-AKT Ser473 and also exhibited additive effects with vemurafenib or AZD6244 in further decreasing p-S6 and p-4EB-P1. These data suggest that S6 and 4EB-P1 are also potential cross-talk points between the AKT and MAPK pathways. Therefore, at least in this case, inhibition of both pathways is necessary to overcome the resistance to vemurafenib and AZD6244. Moreover, the inhibitory effect of RICTOR knockdown on growth of resistant cells suggests that activation of AKT by mTORC2 feedback may play a role in maintenance or even induction of cell growth and therefore can be one of the causes of resistance to MAPK pathway inhibitors. AKT activation by the feedback mechanism could be the cause of growth inducing effect of vemurafenib in resistant cell lines. This feed back mechanism occurs through the induction of mTORC2, which contains RICTOR, and causes higher levels of S473 p-AKT. It should be mentioned that the ability of a combination of MAPK pathway and AKT/mTORC inhibitors to reverse resistance to single agent MAPK inhibitors was not absolute and inefficient for some of the cell lines with primary and acquired cross-resistance to vemurafenib and AZD6244. Growth inhibition assays indicated that combinations of chemical inhibitors of MAPK and AKT pathways can decrease growth rates of some of the vemurafenib resistant cell lines. However these decrease in growth rates of resistant cell lines were not accompanied by the induction of apoptosis in these cell lines, particularly when vemurafenib alone or in combination was used. In the resistant cell lines, inhibition of MEK by AZD6244 could cause some induction cleaved caspase 3 in comparison to the vehicle treated samples. However, levels of cleaved caspase 3 were not increased further by the combination of AZD6244 and inhibitors of AKT pathway. This discrepancy between the growth and apoptosis assays perhaps indicate that these drug combinations may inhibit the growth by mechanisms other than apoptosis or through the ways which do not cause the induction of cleaved caspase 3.

Other investigators have provided convincing evidence that the effects of targeted inhibitors on melanoma cell lines is different in 2-dimension and 3-dimension models [27], with higher resistance to BRAF inhibitors in 3 dimension models mediated by the PI3K/AKT pathway [28]. This was not tested in our studies, and may further underscore the importance of co-targeting both the MAPK and the PI3K/AKT/mTOR pathways for more profound antitumor effects in cells with acquired resistance to single agent BRAF inhibitors. Another possibility to expand on our studies would be the testing of siRNA or an isoform-specific inhibitor of AKT3, which has been previously described to be important in melanoma [29]. The fact that the particular inhibitor used by us (which preferentially blocks AKT1 and AKT2, but at higher concentrations also blocks AKT3) had synergistic effects with vemurafenib or AZD6244 in several cells with cross-resistance to either single agent underscores the promise of co-targeting both pathways as means to treat acquired resistance to BRAF inhibitors.

In conclusion, our data suggest that single agent MEK inhibitor has low activity in vemurafenib-resistant melanoma and perhaps restricted to a subset of cases with secondary oncogenic mutations in NRAS. However, upon progression the addition of an AKT or an mTOR inhibitor to the continued therapy with vemurafenib, or switching to a combination of a MEK inhibitor plus an AKT or an mTOR inhibitor, may provide additional inhibitory activities. Our data strengthens the results from other groups that have previously demonstrated the superior antitumor activity of combining MAPK and PI3K/AKT/mTOR pathway inhibitors in BRAF^V600E^ mutant cell lines [14], [24], [30], by testing this concept in isogenic pairs of sensitive and acquired resistant cell lines, and in cell lines established directly from patients progressing after a response on vemurafenib. Therefore, the elucidation of the molecular mechanisms that result in primary or acquired resistance to vemurafenib and sensitivity to combined MAPK and AKT/mTOR pathway inhibition, would provide useful biomarkers to rationally choose the most appropriate therapy in BRAF^V600E^ mutant melanomas resistant to vemurafenib.

## Supporting Information

Figure S1 **Examples of viability assays at different concentrations of vemurafenib or AZD6244.** Effects of vemurafenib or AZD6244 on cell growth and viability using an MTS assay was determined in the previously established cell line M229 and its *in vitro* acquired resistance M229-AR9 subline.
(TIF)

Figure S2 **Effects of RAPTOR knockdown by siRNAs in combination with either vemurafenib or AZD6244.** The efficiency of siRNA knockdowns and its effect on downstream signaling determined by Western blot analysis of protein lysates (a). M238 parental (b) and M238-AR2 resistant subline (c) were transfected with RAPTOR siRNAs and cultured in increasing concentrations of vemurafenib or AZD6244. The effect of raptor knockdown on resistance and growth inhibition was analyzed after 120 hours by an MTS assay. D in each graph refers to the un-transfected untreated cells and is used as the 100% reference point for all the conditions in the assays.
(TIF)

Figure S3 **Cleaved caspase-3 in sensitive and adaptive resistant cell lines treated with vemurafenib, AZD6244, rapamycin, AKTi.** Cell lines were treated by the solvent (DMSO), 2 µM of vemurafenib, AZD6244, AKTi or 10 nM of rapamycin for 48 hours. Each sample was analyzed by Western blotting using a cleaved caspase-3 (CC3) specific antibody.
(TIF)

Figure S4 **Diagram of pathways and possible cross-talk points involved in survival and resistance of melanoma cell lines.**
(TIF)
