# Supplementary figures and images for: Reversing Melanoma Cross-Resistance to BRAF and MEK Inhibitors by Co-Targeting the AKT/mTOR Pathway
Source: PLoS One. 2011 Dec 14;6(12):e28973. doi: 10.1371/journal.pone.0028973 (PMC3237573; doi:10.1371/journal.pone.0028973)

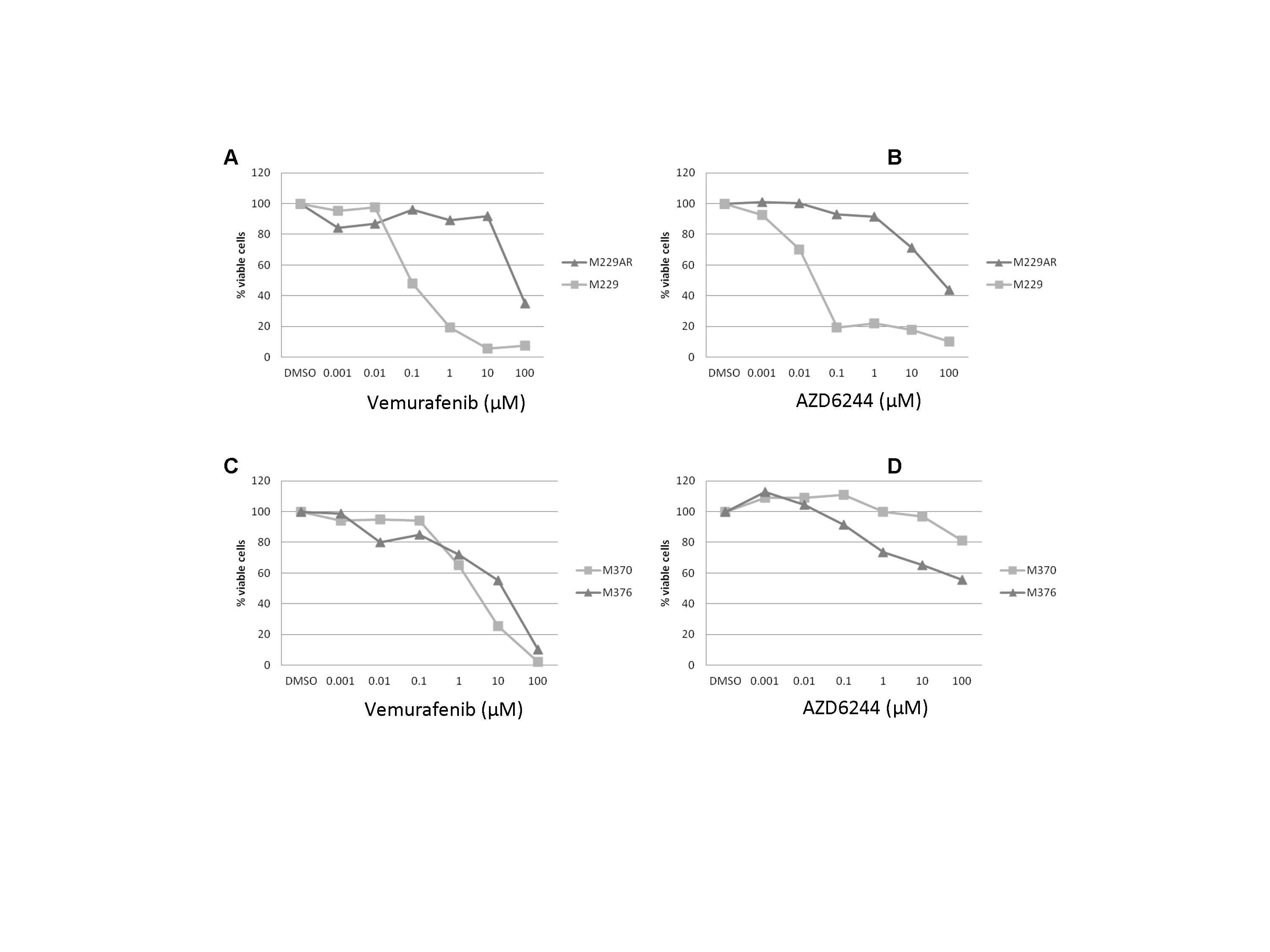

Supplement: Figure S1 — Examples of viability assays at different concentrations of vemurafenib or AZD6244. Effects of vemurafenib or AZD6244 on cell growth and viability using an MTS assay was determined in the previously established cell line M229 and its in vitro acquired resistance M229-AR9 subline. (TIF) [file pone.0028973.s001.tif]

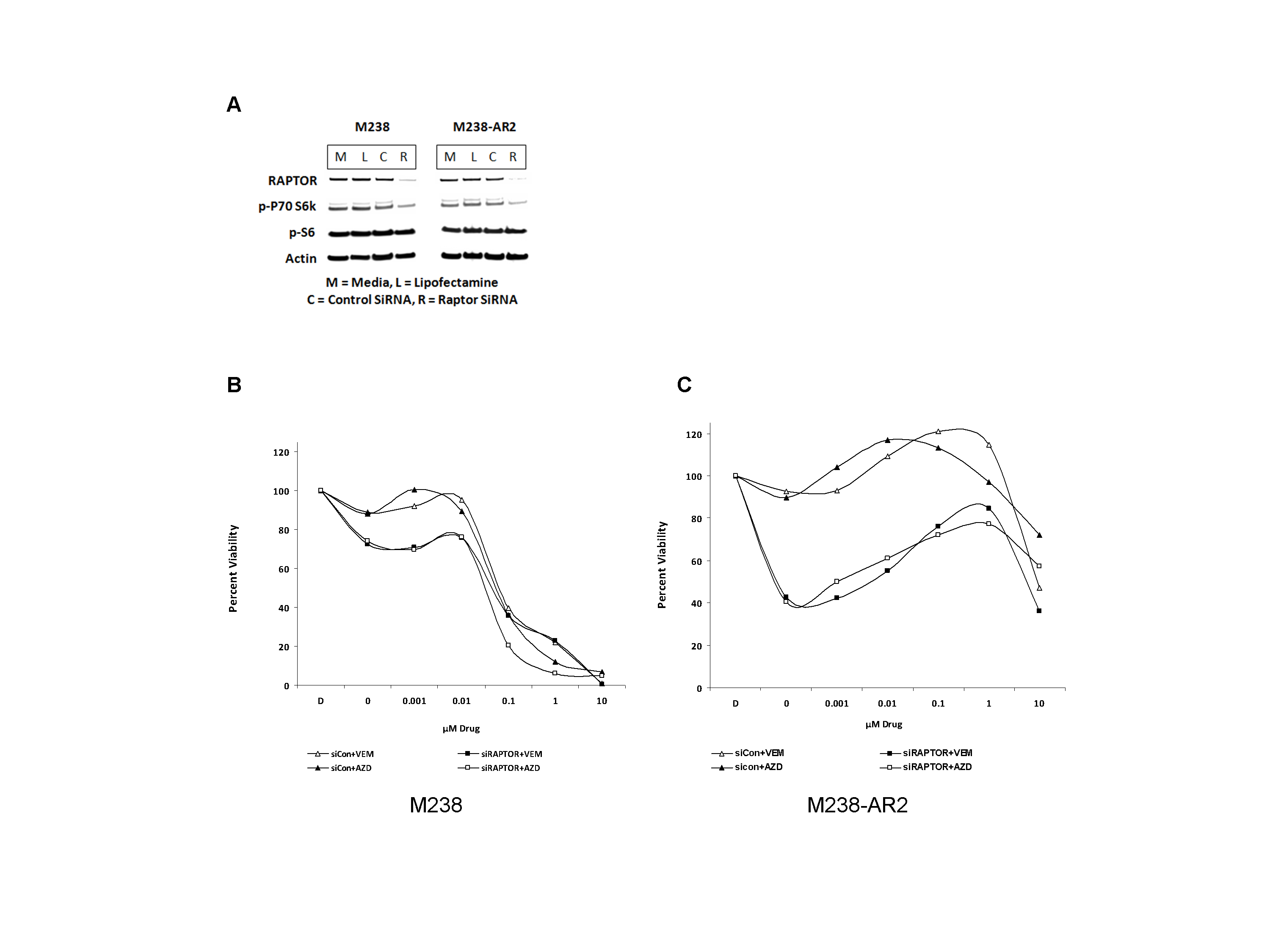

Supplement: Figure S2 — Effects of RAPTOR knockdown by siRNAs in combination with either vemurafenib or AZD6244. The efficiency of siRNA knockdowns and its effect on downstream signaling determined by Western blot analysis of protein lysates (a). M238 parental (b) and M238-AR2 resistant subline (c) were transfected with RAPTOR siRNAs and cultured in increasing concentrations of vemurafenib or AZD6244. The effect of raptor knockdown on resistance and growth inhibition was analyzed after 120 hours by an MTS assay. D in each graph refers to the un-transfected untreated cells and is used as the 100% reference point for all the conditions in the assays. (TIF) [file pone.0028973.s002.tif]

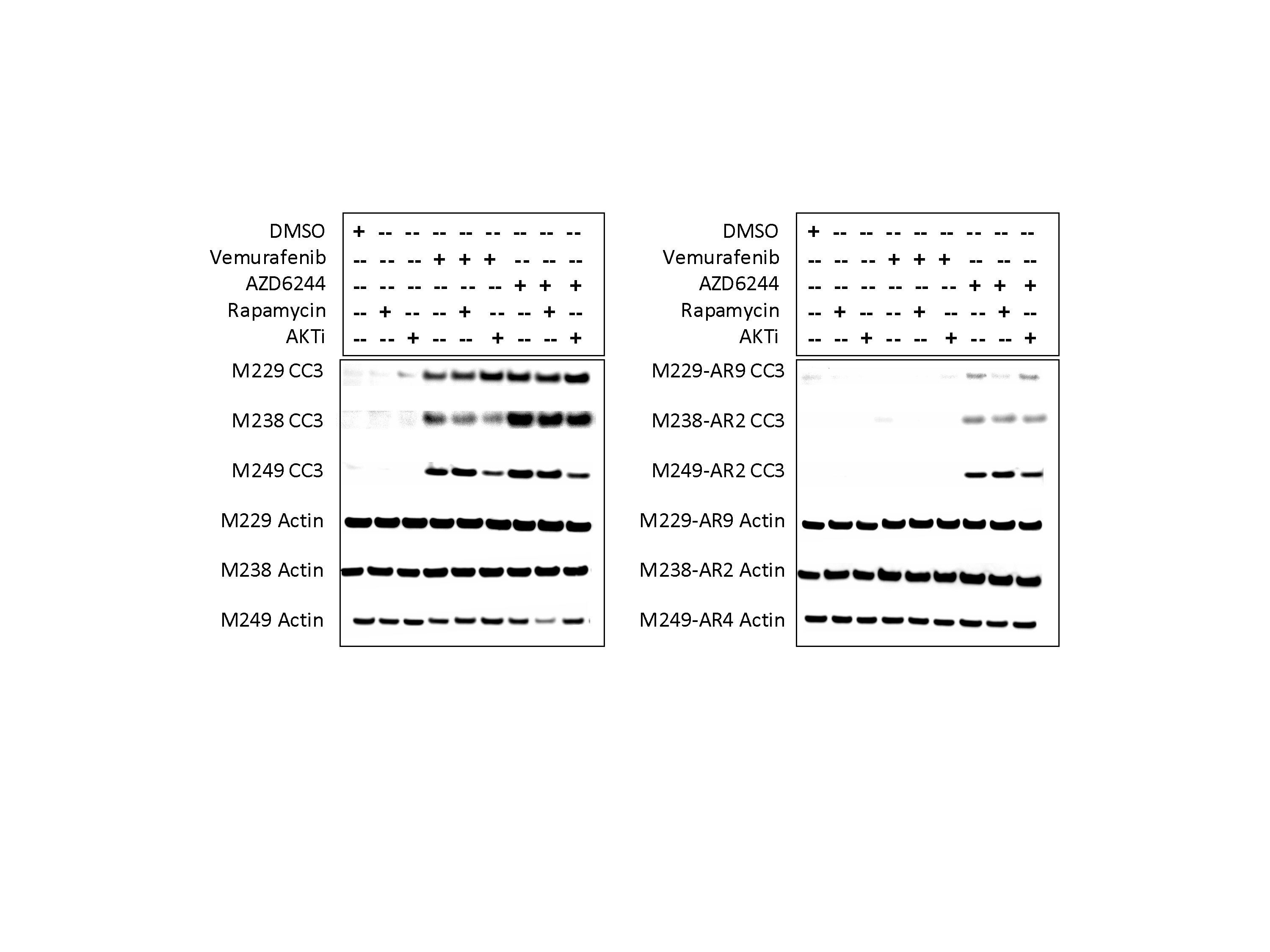

Supplement: Figure S3 — Cleaved caspase-3 in sensitive and adaptive resistant cell lines treated with vemurafenib, AZD6244, rapamycin, AKTi. Cell lines were treated by the solvent (DMSO), 2 µM of vemurafenib, AZD6244, AKTi or 10 nM of rapamycin for 48 hours. Each sample was analyzed by Western blotting using a cleaved caspase-3 (CC3) specific antibody. (TIF) [file pone.0028973.s003.tif]

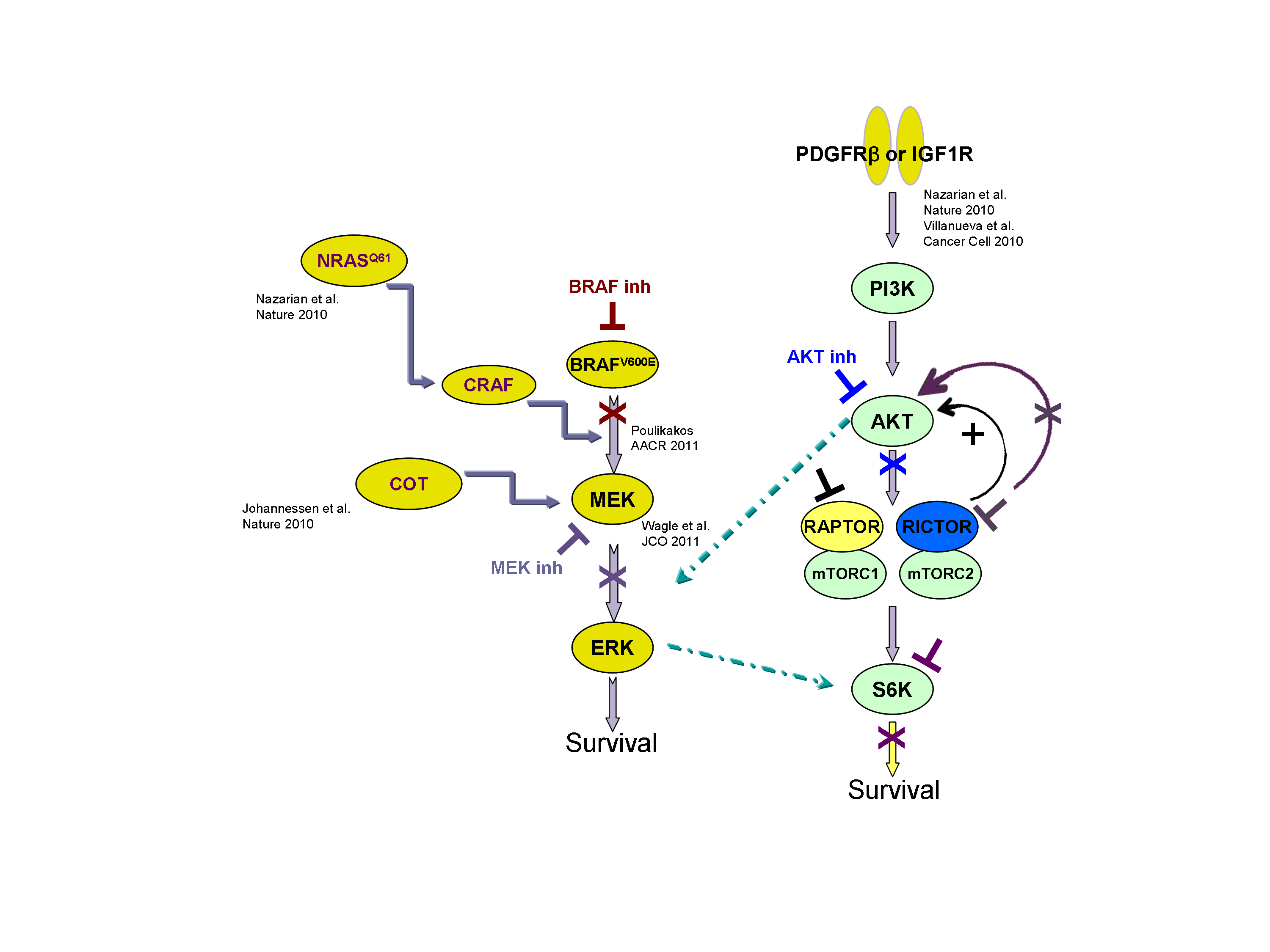

Supplement: Figure S4 — Diagram of pathways and possible cross-talk points involved in survival and resistance of melanoma cell lines. (TIF) [file pone.0028973.s004.tif]
